# Experimental Validation of Normalized Uniform Load Surface Curvature Method for Damage Localization

**DOI:** 10.3390/s151026315

**Published:** 2015-10-16

**Authors:** Ho-Yeon Jung, Seung-Hoon Sung, Hyung-Jo Jung

**Affiliations:** 1 Department of Civil and Environmental Engineering, Korea Advanced Institute of Science and Technology (KAIST), 291 Daehak-ro, Yuseong-gu, Daejeon 305-701, Korea; E-Mail: soulpower@kaist.ac.kr; 2 Agency for Defense Development, Yusong P.O.Box 35, DaeJeon 305-600, Korea; E-Mail: sshgns@gmail.com

**Keywords:** modal flexibility, ULS, ULS curvature, NULS, normalization, damage localization

## Abstract

In this study, we experimentally validated the normalized uniform load surface (NULS) curvature method, which has been developed recently to assess damage localization in beam-type structures. The normalization technique allows for the accurate assessment of damage localization with greater sensitivity irrespective of the damage location. In this study, damage to a simply supported beam was numerically and experimentally investigated on the basis of the changes in the NULS curvatures, which were estimated from the modal flexibility matrices obtained from the acceleration responses under an ambient excitation. Two damage scenarios were considered for the single damage case as well as the multiple damages case by reducing the bending stiffness (*EI*) of the affected element(s). Numerical simulations were performed using MATLAB as a preliminary step. During the validation experiments, a series of tests were performed. It was found that the damage locations could be identified successfully without any false-positive or false-negative detections using the proposed method. For comparison, the damage detection performances were compared with those of two other well-known methods based on the modal flexibility matrix, namely, the uniform load surface (ULS) method and the ULS curvature method. It was confirmed that the proposed method is more effective for investigating the damage locations of simply supported beams than the two conventional methods in terms of sensitivity to damage under measurement noise.

## 1. Introduction

Regular structural health monitoring is imperative for preventing catastrophic structural failure, for increasing the cost-effectiveness of maintaining existing structures, and for maintaining the serviceability of existing structures. During the past three decades, vibration-based damage detection methods based on various dynamic properties, such as the natural frequencies, mode shapes, mode shape curvatures, and modal flexibility matrices, have seen significant development. The basic concept of these vibration-based methods is that changes in the boundary and physical characteristics of structures lead to changes in the structural modal parameters, that is, in the natural frequencies and mode shapes. Thus, damage localization can be performed by comparing the characteristics obtained from the modal parameters before and after damage. In the case of modal parameter-based damage detection techniques, the system identification method is crucial for obtaining the structural modal parameters. In order to be able to detect damage with precision, various signal processing and system identification techniques have been proposed. Nagarajaiah, Nagarajaiah and Basu, and Worden *et al.* [1,2,3] have presented a number of time- and frequency-domain identification methods. Recently, Yang and Nagarajaiah [4] reported time-frequency blind source separation for an output-only modal identification technique. Further, excellent reviews of the available vibration-based damage detection methods can be found in Doebling *et al.*, Sohn *et al.*, and Fan and Qiao [5,6,7].

The modal flexibility method in particular, has received considerable attention as one of the most promising damage descriptors due to its high sensitivity to damage. The first reported study based on this method was by Pandey and Biswas, who used the changes in the modal flexibility matrices [8]. A number of studies have utilized the uniform load surface (ULS) [9], the ULS curvature [10,11], and the damage locating vector (DLV) based on the changes in the modal flexibility matrices while employing an intact finite element model [12,13,14,15]. The ULS method exhibits a smaller truncation effect and is relatively insensitive to experimental errors. However, it is difficult to distinctively localize multiple damages using this method. On the other hand, the ULS curvature method is highly recommended for the localization of multiple damages, given it high sensitivity to closely distributed damages. However, it is vulnerable to noise during curvature estimations and insensitive to damage near regions with small moments. The DLV method can identify damage in elements having negligible internal forces but is dependent on the accuracy of the modal flexibility matrix of the intact structure.

Recently, the normalized uniform load surface (NULS) curvature method [16] has been proposed to overcome the above-mentioned drawbacks of the existing modal flexibility-based damage detection methods. The NULS curvature method uses the normalization technique to identify damage near regions with small moments. However, the one previous study that employed this method [16] was limited to the “proof-of-concept” level and involved only numerical investigations. Further, the relationship between the changes in the NULS curvatures and the damage incurred was demonstrated only analytically. Finally, the numerical simulations were performed while taking into account the measurement noise. Thus, it is necessary to validate the feasibility of the NULS curvature method under more practical conditions.

In this study, the performance of the NULS curvature method was investigated experimentally using a lab-scale simply supported beam model. In order to show the superiority of the NULS curvature method, damage detection was also performed using two well-known methods, namely, the ULS method [9] and the ULS curvature method [10,11], and the results obtained using the three methods were compared.

## 2. Theory

### 2.1. Uniform Load Surface (ULS) Method

The modal flexibility matrix, G_m_, can be expressed using the lower mode, m, as follows:

(1) $$G_{m}=\Phi_{m}\Lambda_{m}^{-1}\Phi_{m}^{Τ}=\sum_{i=1}^{m}\frac{\Phi_{i}\Phi_{i}^{Τ}}{\omega_{i}^{2}}$$

where $\Lambda_{m}=\left[\backslash \omega_{i}^{2}\backslash \right]$, for which $\omega_{i}$ is the *i*-th structural natural frequency, *i* = 1, 2, …, m; $\Phi_{m}=\left\{\varphi_{1},\varphi_{2}\cdots ,\varphi_{m}\right\}$; and $\varphi_{i}$ is the *i*-th mass-normalized mode shape [17,18,19,20,21]. After calculating the modal flexibility matrices, the deflections of the structure under the unit load vector can be computed by simple matrix multiplication as

(2) $$u=\left[\begin{array}{ccccc} {\text{G}}_{\text{11}} & \cdots & {\text{G}}_{\text{1i}} & \cdots & {\text{G}}_{\text{1n}}\\ \vdots & \ddots & \vdots & ⋰ & \vdots \\ {\text{G}}_{\text{i1}} & \cdots & {\text{G}}_{\text{ii}} & \cdots & {\text{G}}_{\text{in}}\\ \vdots & ⋰ & \vdots & \ddots & \vdots \\ {\text{G}}_{\text{n1}} & \cdots & {\text{G}}_{\text{ni}} & \cdots & {\text{G}}_{\text{nn}} \end{array}\right]\left\{\begin{array}{c} 1\\ \vdots \\ 1\\ \vdots \\ 1 \end{array}\right\}$$

where u is the deflection obtained from the modal flexibility under the ULS, and G_ij_ is the (i, j)-th component of the modal flexibility matrix.

Damage localization can be performed by comparing the deflections for the intact case and the damage case under the ULS. As mentioned previously, the ULS method exhibits a smaller truncation effect and is relatively insensitive to experimental errors [9]; however, it is difficult to distinctively localize multiple damages using this method.

### 2.2. ULS Curvature Method

The curvature of a structure based on the mechanics of materials theory can be estimated as

(3) $${u^{\prime }}^{\prime }=\frac{{\text{d}}^{\text{2}}u}{{\text{dx}}^{\text{2}}}=\frac{M}{EI}$$

where ${u^{\prime }}^{\prime }$ is the curvature at the section, M is the bending moment, E is the elasticity modulus, and I is the moment of inertia at the section. Therefore, the change in the ULS curvature at the *i*-th element induced by damage occurring at the *i*-th element can be expressed as

(4) $$\frac{\Delta M(i)}{EI}=\left(\frac{\Delta u_{i+1}-2\Delta u_{i}+\Delta u_{i-1}}{l_{i}^{\;2}\;}\right)$$

where $\frac{\Delta M(i)}{EI}$ is the change in the ULS curvature before and after damage at the *i*-th element; $\Delta u_{i}=u_{i}^{D}-u_{i}^{I}$ is the change in the deflection at the *i*-th element, as estimated from the modal flexibility under the ULS; $u_{i}^{I}$ and $u_{i}^{D}$ are the deflections of the intact and damaged structures at the *i*-th element under the ULS, respectively; and *l*_i_ is the length of an element.

As mentioned earlier, the ULS curvature method can localize multiple damages owing to its high sensitivity to closely distributed damages [10,11]. However, the method is vulnerable to noise during curvature estimations and insensitive to damage near regions with small moments.

### 2.3. Normalized Uniform Load Surface (NULS) Curvature Method

Consider a beam-type structure that follows the Bernoulli-Euler beam theory and undergoes column damage, ΔK. Then, the forces, ΔF, attributable to the damage, which would be proportional to the stiffness reduction, can be obtained as follows [22,23]:

(5) $$\Delta F=\Delta Ku_{e}=\left\{\begin{array}{c} 0\\ \alpha_{e}f_{e}\\ 0 \end{array}\right\}$$

where ΔK = diag(0, $\alpha_{e}$k_e_, 0), $\alpha_{e}$ is the damage ratio, 0 < $\alpha_{e}$ < 1, k_e_ is the elementary stiffness matrix representing the intact state of the damaged system, and f_e_ = k_e_u_e_ is the stress resultant of the element in the intact system. Then, the changes in the ULS curvatures can be expressed by following equation:

(6) $$\mathrm{Changes in ULS curvature}=\left\{\frac{M(x)+\Delta M(i)}{EI}\right\}-\left\{\frac{M(x)}{EI}\right\}=\frac{\Delta M(i)}{EI}$$

where M(x) is the internal force under the ULS along the *x*-axis, and $\frac{\Delta M(i)}{EI}$ is the change in the ULS curvature at the *i*-th element.

The changes in the ULS curvature are insensitive to damage in regions with small moments, owing to M(*i*) in ΔM(*i*). In order to solve this problem, the NULS curvature method was proposed; in this method ΔM(*i*) is divided by M(*i*) [16]. The NULS curvature method also compares the ratio of the change in moment at the measured points before and after damage. By comparing the ratio of the change in moment, the sensitivity to damage in the case of small moments can be increased. Thus, the changes in the NULS curvature can be calculated as

(7) α(i) =ΔM(i)M(i)=(Δui+1−2Δui+Δui−1li 2 ui+1I−2uiI+ui−1Ili 2)

where α(i) is the change in the NULS curvature at the *i*-th element, $\Delta u_{i}=u_{i}^{D}-u_{i}^{I}$ is the damage-induced deflection caused by damage under the ULS, and $u_{i}^{I}$ and $u_{i}^{D}$ are the displacements estimated from the modal flexibilities of the intact and damaged structures, respectively.

The ULS method encounters difficulties in localizing damage near the structural boundary, as the associated deflections are relatively small. Further, it is difficult to distinctively localize multiple damages. The ULS curvature method is based on the changes in the structural moment before and after damage. Therefore, it can localize the location of damage even in the case of multiple damages. However, the ULS curvature method is insensitive to damage near regions with small moments. The proposed NULS curvature method overcomes this insensitivity to damage at small deflections and regions with moment change. The proposed method involves comparing the ratio of the change in the ULS curvature before and after damage. Hence, the ratio of the change in the ULS curvature is sensitive to damage even in the case of regions with small moments and deflections.

### 2.4. Damage Localization by NULS Curvature Method under Noisy Measurements

Unexpected changes in the NULS curvature may occur at intact regions owing to inevitable measurement noise. Therefore, statistical approaches are preferred for preventing false-positive or false-negative damage detections. In this study, a normalized outlier index, NF(*i*), of the changes in the NULS curvature was employed for identifying the damage locations:

(8) $$NF\text{(}i\text{)}=\frac{\bar{\bar{M}}\text{(}i\text{)}}{{\bar{M}}_{0}\text{(}i\text{)}}\frac{M\text{(}i\text{)}-{\bar{M}}_{0}\text{(}i\text{)}}{\sigma \text{(}M_{0}\text{(}i\text{))}}=\kappa \text{(}i\text{)}Z\text{(}i\text{)}$$

where $\bar{\bar{M}}\text{(}i\text{)}$ is the mean value of the ULS curvatures at the *i*-th sensor node in the current state. ${\bar{M}}_{0}\text{(}i\text{)}$ is the mean value of the ULS curvature at the *i*-th sensor node, and $\sigma \text{(}M_{0}\text{(}i\text{))}$ is the standard deviation of the ULS curvatures for the intact system. $\kappa \text{(}i\text{)}=\frac{\bar{\bar{M}}\text{(}i\text{)}}{{\bar{M}}_{0}\text{(}i\text{)}}$ is the constant for normalizing the outlier index. $Z\text{(}i\text{)}=\frac{M\text{(}i\text{)}-{\bar{M}}_{0}\text{(}i\text{)}}{\sigma \text{(}M_{0}\text{(}i\text{))}}$ is the outlier index based on the standard normal distribution. NF(*i*) is a normalized outlier index, that is, a damage index, based on the statistical approach. As mentioned above, because of the inevitable measurement noise, statistical approaches are preferred. If the value of NF(*i*) is higher than the threshold value, structural damage is localized at the *i*-th sensor node.

When a damage alert is issued, the damage location can be evaluated using following condition:

Damage is located at the i-th element if NF(i) > NFThreshold for any i
(9)

**Figure 1:**
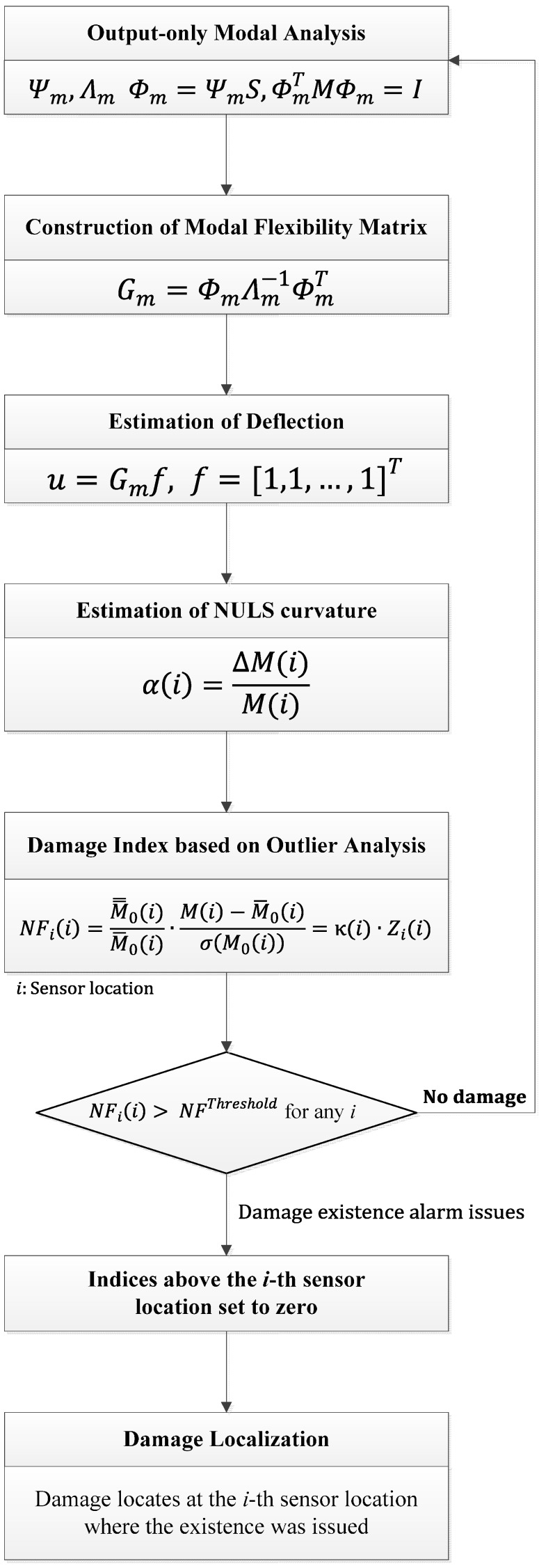
Flow chart of the proposed damage detection method.

The damage detection procedure is shown in Figure 1.

## 3. Experimental Validation of the NULS Curvature Method

### 3.1. Numerical Simulations as a Preliminary Step

**Figure 2:**
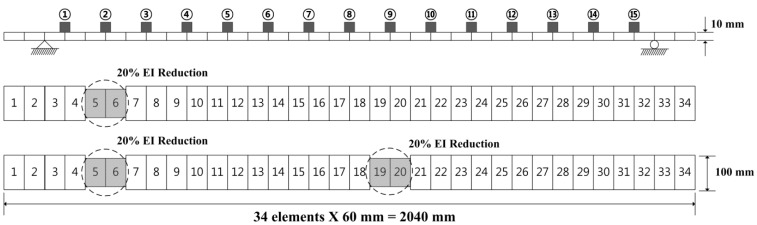
Schematics of the simply supported beam model and its damage scenarios.

Numerical simulations were performed as a preliminary step using a model of the lab-scale simply supported beam; the model is shown in Figure 2 and the material properties are listed in Table 1. The test structure was modeled using beam elements. It was composed of a total of 34 elements and was made using MATLAB. The damages were simulated by reducing the bending stiffness (*EI*) of the affected elements. Further, two categories of damages were considered, as shown in Figure 1. These were single damage (DC1) and multiple damages (DC2). The changes induced in the structural modal parameters under these damages are shown in Table 2. The natural frequencies were found to decrease by 0.064%–0.654%; in contrast, the modal assurance criterion (MAC) values did not change much. The results of the numerical simulations showed that the NULS curvature method could successfully identify single as well as multiple damages.

**Table 1:** Properties of the structural model used.

Parameters	Value
Damping ratio	0.005
Mass density (ρ)	7850 kg m^−3^
Poisson’s ratio (υ)	0.28
Elasticity modulus (*E*)	200 GPa
Length (*L*)	2.04 m
Width (*w*)	100 mm
Thickness (*t*)	10 mm

**Table 2:** Changes in modal parameters due to damages.

Case	First Mode			Second Mode		
	*f*_1_ (Hz)	*Δ f*_1_/*f*_1_ (%)	MAC	*f*_2_ (Hz)	*Δ f*_2_/*f*_2_ (%)	MAC
IC	6.1018	-	1.0000	24.273	-	1.0000
DC1	6.0979	−0.064%	0.9999	24.217	−0.230%	0.9999
DC2	6.0619	−0.654%	0.9999	24.193	−0.328%	0.9999

**Figure 3:**
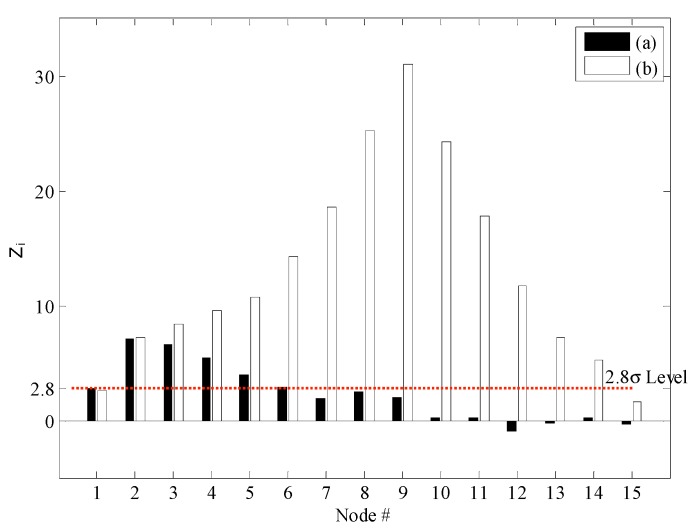
Results of damage detection using the ULS method: (**a**) DC1 and (**b**) DC2.

**Figure 4:**
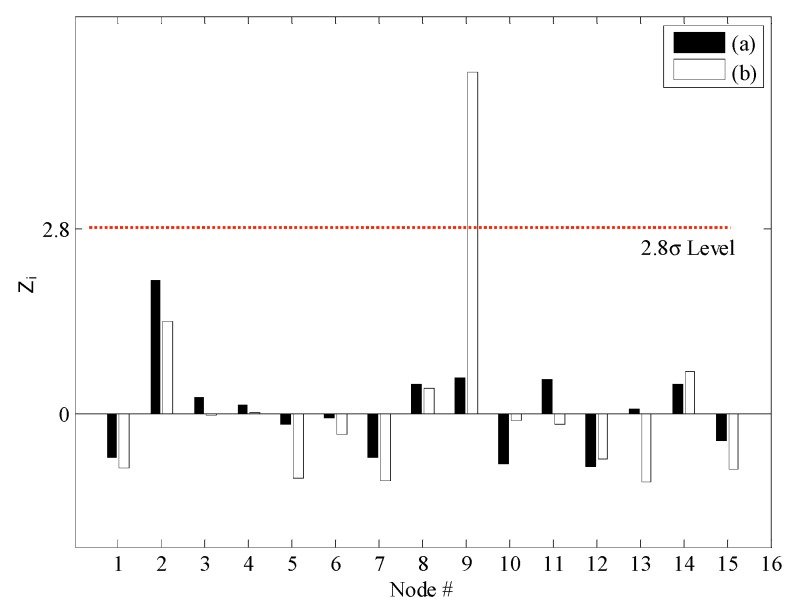
Results of damage detection using the ULS curvature method: (**a**) DC1 and (**b**) DC2.

**Figure 5:**
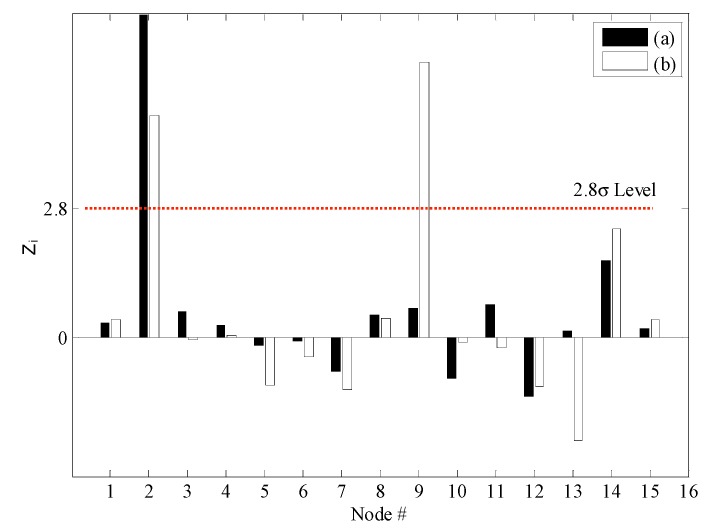
Results of damage detection using the NULS curvature method: (**a**) DC1 and (**b**) DC2.

The outlier index was applied and the threshold value of the damage index is chosen by the user. In this study, it was set at 2.8, based on the standard normal distribution. The location of the damage is the peak node of the change in the ULS. As shown in Figure 3, for DC1, the peak node was the 3rd sensor node. However, the actual location of the damage was the 2nd sensor node. Therefore, a false damage alarm occurred in the case of single damage. Next, for DC2, the peak nodes for the change in the ULS were the 2nd and 9th nodes. The change in the ULS at the 9th sensor node was distinctive and the node could be considered as the damage node. However, the changes at the 2nd and 3rd sensor nodes were almost similar. Thus, it was hard to define the locations of the damages. The damage detection performance of the ULS method is poor, especially for damages located near boundaries. As shown in Figure 4, for DC1, the ULS curvature method did not yield the location of the damage. All the values were lower than the threshold level of 2.8. For DC2, the change in the ULS curvature occurred at the 9th node only. Further, it was clear that the element at the 9th node was the damaged element. Even though this was a multiple damages case, just one damaged element was found. Finally, as shown in Figure 5, in the case of the NULS curvature method, the damaged element was found at the 2nd node for DC1. For DC2, two damage elements were found, at the 2nd node and the 9th node. Thus, the NULS curvature method yielded the damage locations for both DC1 and DC2.

### 3.2. Experimental Results

**Figure 6:**
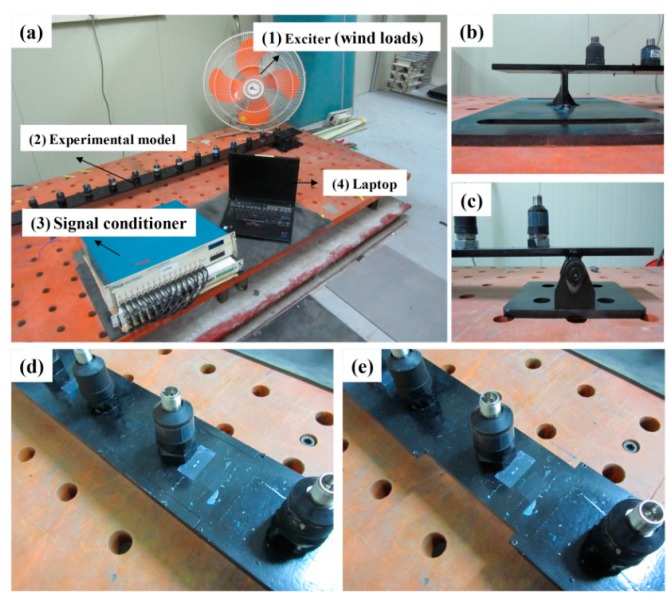
Experimental setup and damage scenarios: (**a**) experimental setup; (**b**) roller support; (**c**) pin support; (**d**) intact case; and (**e**) damage case.

Experimental validations were performed using a simply supported beam; the structure was supported by a roller and a pin [24], as shown in Figure 6. The structural characteristics of this structure were essentially the same as those used in the numerical simulations (see Table 1). An ambient vibration test was performed under excitation using wind loads, and 15 accelerometers (PCB 393B12) were installed on the surface of the structure at similar intervals (12 cm). Two damage scenarios for the multiple damages case as well as the single damage case were considered, as shown in Figure 2.

**Figure 7:**
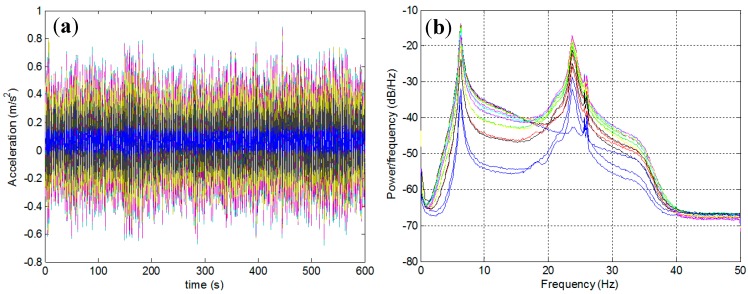
Typical time-domain signals (**a**) and their power spectra (**b**) (for the intact case).

Experiments lasting 10 min were performed for each intact/damage case. The sampling frequency was 100 Hz, and an anti-aliasing filter (35 Hz) was used on the raw measurement data. The typical time-domain signals and their power spectra are depicted in Figure 7. In order to ensure that the uncertainties in the modal parameters and damage detection results were accounted for, the measurement was repeated eight times for each intact/damage case. One crucial advantage of the modal flexibility is that it can be reliably estimated using a few lower modes. The number of measurable modes in the field is limited owing to the rigidity of the structures and because of the low excitation level. Thus, in these experiments, the two lower modes were used to calculate the modal flexibility matrices for each intact/damage case.

**Figure 8:**
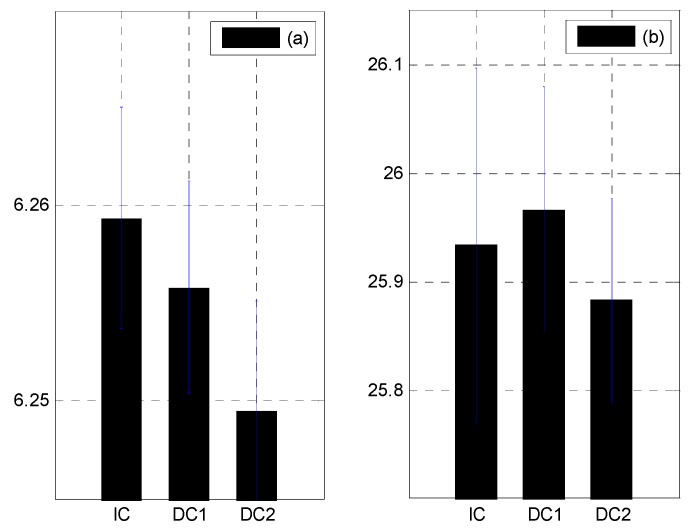
Changes in the natural frequencies and standard deviation owing to damages: (**a**) the first mode and (**b**) the second mode.

**Figure 9:**
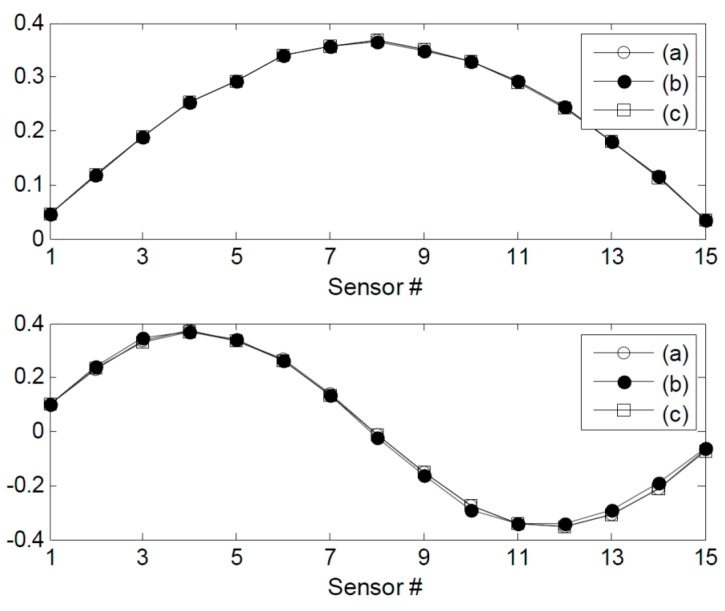
Mode shapes of (**a**) IC (**b**) DC1, and (**c**) DC2.

**Figure 10:**
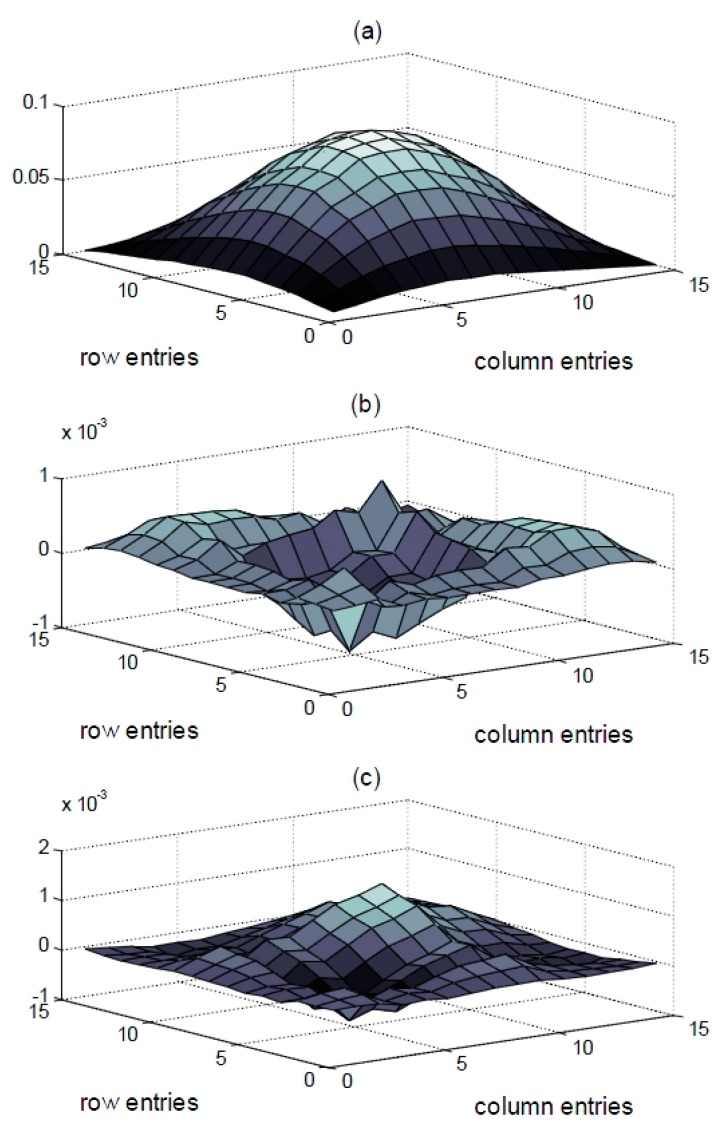
Plots of the matrices for the (**a**) flexibility of the intact specimen (**b**) differences in the flexibilities corresponding to IC and DC1, and (**c**) differences in the flexibilities corresponding to IC and DC.

The stochastic subspace identification method [25] was used to identify the modal parameters of the test structure. The changes in the modal parameters due to the damages are shown in Table 3 and Figure 8 and Figure 9. The mass-normalized mode shapes were obtained from the system mass matrix. The natural frequencies decreased by 0.0567%–0.1960%. However, there were no observable changes in the MAC values. The second natural frequency for DC1 increased even though damage had been done. This may be attributed to the effects of the measurement noise and the decrease in the structural mass. However, since the first natural frequency was dominant over the others when calculating the structural flexibility matrix, this increase was negligible. The three-dimensional intact flexibility matrix is plotted in Figure 10a, while the differences in the flexibility matrices of the intact and damaged structures are plotted in Figure 10b,c. It is apparent that the damage locations could not be identified clearly by introducing the baseline modal parameters of the structure.

**Table 3:** Changes in the modal parameters due to damage.

Case	First Mode			Second Mode		
	*f*_1_ (Hz)	*Δ f*_1_/*f*_1_ (%)	MAC	*f*_2_ (Hz)	*Δ f*_2_/*f*_2_ (%)	MAC
IC	6.2593	-	1.0000	25.934	-	1.0000
DC1	6.2558	−0.0567%	0.9999	25.967	0.1273%	0.9992
DC2	6.2495	−0.1580%	0.9999	25.883	−0.1960%	0.9999

**Figure 11:**
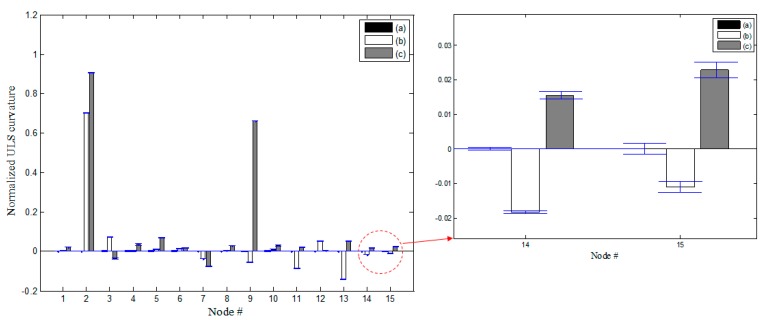
Changes in the NULS curvatures and its magnified plot at node 14, 15 with a deviation of 1σ: (**a**) IC, (**b**) DC1 from IC, and (**c**) DC2 from IC.

**Figure 12:**
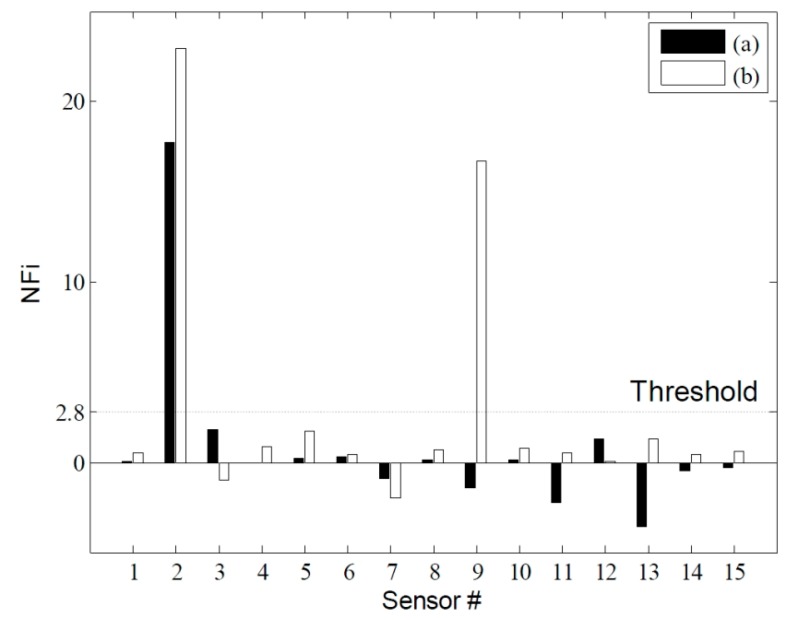
Damage localization based on the normalized outlier index: (**a**) DC1 from IC and (**b**) DC2 from IC.

Figure 11 shows the changes in the NULS curvatures corresponding to a deviation of 1σ. In the case of DC1, damage at the 2nd sensor node could be identified clearly on the basis of the changes in the NULS curvatures. Further, in the case of DC2, multiple damage locations could be localized successfully at the locations of the 2nd and 9th sensors. However, small changes were also observed in the NULS curvatures at the undamaged locations, owing to measurement noise. In order to reduce the rate of false-positive and false-negative damage detections to less than 0.5%, the normalized outlier index, *NF*(*i*), which had a threshold value 2.8, was used; this was on the basis of the normal distribution. Figure 12 shows the results of the damage localizations performed using *NF*(*i*). In the case of DC1, the damage location could be identified clearly as the location of the 2nd sensor. In the case of DC2, the damage locations could also be correctly identified using *NF*(*i*), and no false-positive or false-negative detections were made.

### 3.3. Comparative Study

The damage detection performance of the NULS curvature method was compared against those of two other well-known damage detection methods: (1) the ULS method [9] and (2) the ULS curvature method [10,11]. In the case of the ULS method, when the existence of damage is indicated by the damage index, the peak node corresponding to the changes in the deflections under the ULS is considered the damage location. In the case of the ULS curvature method, damage is assumed to be located at the *i*-th sensor location, if the changes in ULS curvature exceed the threshold for any *i*.

**Figure 13:**
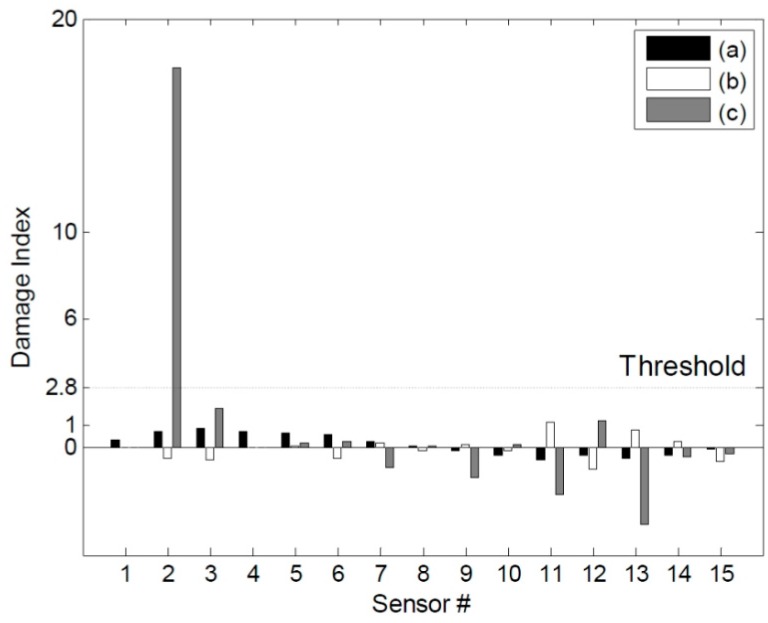
Estimations of the damage location for DC1 using the three different methods: (**a**) the ULS method, (**b**) the ULS curvature method, and (**c**) the NULS curvature method.

**Figure 14:**
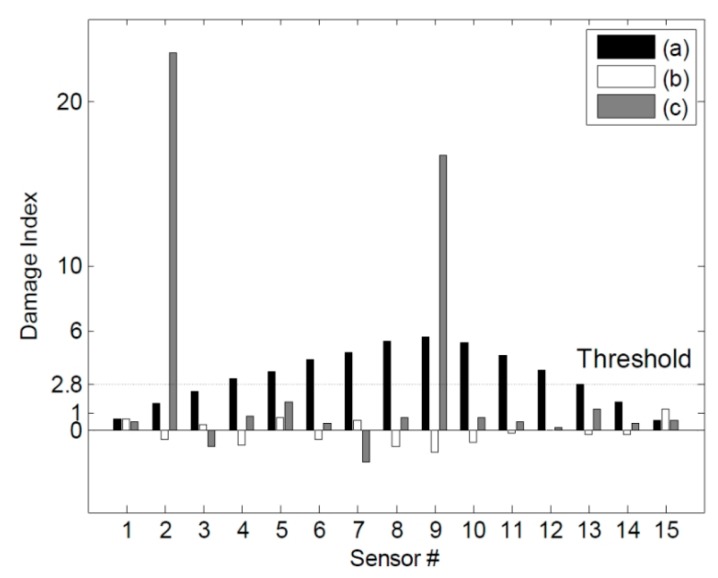
Estimations of the damage locations for DC2 using the three different methods: (**a**) the ULS method; (**b**) the ULS curvature method; and (**c**) the NULS curvature method.

Figure 13 shows the damage indices for DC1 corresponding to a threshold value of 2.8. The damage at the 2nd sensor location could be identified successfully using the NULS curvature method. In the case of the other two methods, however, damage localization was not possible, because of the presence of measurement noise and since the small moments near the roller support had an adverse effect on the ability to detect damage. Figure 14 shows the damage indices for DC2 for the same threshold value. In the case of the ULS method, the damage at the 9th sensor location could be identified clearly; however, that at the 2nd sensor location could not. Further, in the case of the ULS curvature method, it was difficult to localize all the damage locations, given the noise vulnerability of this method. On the other hand, the NULS curvature method could successfully identify all the damage locations.

The comparative study showed that the NULS curvature method could identify the damages clearly without generating any false positives or false negatives; this was true for all the damage cases. In contrast, the ULS method and the ULS curvature method failed to identify the damage locations. In particular, the ULS curvature method could not identify any damages, given its vulnerability to noise. It was clearly demonstrated that the NULS curvature method performed better at localizing damages than the two conventional modal flexibility based methods and showed both higher sensitivity to damage and lower vulnerability to measurement noise.

## 4. Conclusions

In this study, we experimentally validated the NULS curvature method, which has been developed recently for localizing damage in beam-type structures and is based on the Bernoulli–Euler beam theory. The normalization technique allowed for more sensitive and robust damage localization. In this study, the damage locations of a lab-scale simply supported beam model were numerically and experimentally investigated on the basis of the changes in the NULS curvatures; these were estimated from the modal flexibility matrices obtained from the acceleration responses under an ambient excitation. Two damage scenarios were considered for the multiple damages case as well as the single damage case by reducing the bending stiffness (*EI*) of the affected element(s). After numerical simulations had been performed using MATLAB as a preliminary step, a series of experimental tests were carried out, in order to validate the feasibility of the NULS curvature method. It was found that the damage locations could be identified successfully without any false-positive or false-negative detections on the basis of the normalized outlier index. In order to confirm the superiority of the NULS curvature method, damage detections were also performed using two well-known modal flexibility-based damage detection methods, namely, the ULS method and the ULS curvature method. However, these two methods failed to identify the damage locations. The ULS curvature method, in particular, could not localize any damages, owing to its vulnerability to noise. Therefore, it can be concluded that the NULS curvature method is more effective for investigating the damage locations of simply supported beams than are the two other conventional methods in terms of sensitivity to damage and robustness against measurement noise.
